# The appropriate use of radiography in clinical practice: a report of two cases of biomechanical versus malignant spine pain

**DOI:** 10.1186/1746-1340-14-8

**Published:** 2006-05-30

**Authors:** Roger Kevin Pringle, Lawrence H Wyatt

**Affiliations:** 1 Spine Center/Orthopedics, Kelsey-Seybold Medical Group, Houston, Texas, USA; 2 Division of Clinical Sciences, Texas Chiropractic College, Pasadena, Texas, USA

## Abstract

**Background:**

To describe the evaluation, treatment, management and referral of two patients with back pain with an eventual malignant etiology, who were first thought to have a non-organic biomechanical disorder.

**Clinical features:**

The study was a retrospective review of the clinical course of two patients seen by a chiropractor in a multi-disciplinary outpatient facility, who presented with what was thought to be non-organic biomechanical spine pain. Clinical examination by both medical and chiropractic physicians did not indicate the need for radiography in the early course of management of either patient. Upon subsequent re-evaluation, it was decided that certain clinical factors required investigation with advanced imaging.

In one instance, the patient responded to conservative care of low back pain for nine weeks, after which she developed severe pain in the pelvis. In the second case, the patient presented with signs and symptoms consistent with uncomplicated musculoskeletal pain that failed to respond to a course of conservative care. He was referred for medical therapy which also failed to relieve his pain. In both patients, malignancy was eventually discovered with magnetic resonance imaging and both patients are now deceased, resulting in an inability to obtain informed consent for the publication of this manuscript.

**Conclusion:**

In these two cases, the prudent use of diagnostic plain film radiography did not significantly alter the appropriate long-term management of patients with neuromusculoskeletal signs and symptoms. The judicious use of magnetic resonance imaging was an effective procedure when investigating recalcitrant neuromusculoskeletal pain in these two patients.

## Background

Neuromusculoskeletal (NMS) complaints are one of the most common reasons for physician visits around the world. In addition, nearly all patients who visit doctors of chiropractic present with neuromusculoskeletal complaints. [1] Most of these NMS complaints are 'non-organic' or 'non-specific' in etiology, [1] although serious causes of spine pain such as malignancy, infection and acute fracture are uncommonly found. [2]

Plain film radiography is a staple diagnostic test in the evaluation of spine pain, with patients often being x-rayed on the initial visit, and occasionally re-x-rayed as a follow up procedure. In particular, chiropractors often take radiographs for medico-legal reasons and for use as a screening tool. [3] The rate of radiography performed by doctors of chiropractic in the United States has decreased since the release of the 1998 Job Analysis of Chiropractic from the National Board of Chiropractic Examiners. [3] Yet, many contemporary guidelines regarding the use of radiography in back pain patients suggest that radiography is over-utilized in all health care disciplines, the chiropractic profession included. [4] Also, chiropractors have generally not followed evidence-based guidelines for the use of radiography. [5] However, recent evidence suggests that chiropractors can be trained to use evidence-based guidelines when making decisions about radiography as a diagnostic tool. [6]

Managing low back pain (LBP) is a costly endeavor. An important contributor to the high cost is the use of radiography for assessing patients with acute LBP. In the United States, the annual cost of radiography of the low back was estimated at $500 million in 1991. [7] Interestingly, most of these patients have normal lumbar spine radiographs or age-related degenerative changes that do not correlate with the presence, absence, or severity of pain. [7-11] In some instances, the use of plain film radiography may actually be associated with poorer clinical outcomes. A recent randomized controlled trial suggested that patients with LBP who had radiography experienced decreased functioning, more severe pain, or worse overall health status compared with a control group. [1]

Potential risks associated with spine radiography have also been identified. Because of the close proximity of the reproductive organs for example, lumbar spine radiography results in one of the highest cumulative doses of radiation to the gonads. [8] This exposure increases the risk of cell mutation and cancer in this highly susceptible tissue. [12] According to the International Commission on Radiology Protection, five malignancies are induced per one million persons exposed to lumbar spine radiographs, [13] and in Britain, the National Radiation Protection Board estimates that 19 lives are lost each year because of unnecessary lumbar spine radiographs. [14]

Despite these findings, the majority of doctors of chiropractic say that they would utilize radiography in patients with uncomplicated back pain without the presence of red flags such as high fever and the like. [15,16] Some chiropractors continue to take full-spine radiographs on patients, regardless of symptoms. [5]

No diagnostic test should be ordered unless there is a strong likelihood that the results of that test, either positive or negative, will have an impact on the treatment or prognosis for a patient.

For these varied reasons, diagnostic radiography in the management of spine pain should be used judiciously and it should be based on best practices information. Best practices is a process, which includes an oft-updated document, that reviews the current evidence regarding clinical procedures and helps the clinician provide the best care available to patients by accurately interpreting that evidence and making those interpretations available to clinicians in a readily useable format.

This retrospective case review outlines two cases where best practices were used in the decision making process regarding radiography of the spine in patients presenting to a chiropractor in a multi-disciplinary practice setting. In both cases, the patients were eventually diagnosed with malignancy, but the initial decision to not perform radiography did not have a substantial negative impact on these patients' clinical outcomes.

## Case presentations

One author (RKP) retrospectively reviewed the charts, and both authors as well as a staff radiologist at the facility where the patient was seen, reviewed the diagnostic imaging studies, of two patients who were treated for spine pain in a multi-disciplinary outpatient clinic in Houston, Texas in 2003. There was no attempt at randomization. The patients were a 54-year old African American female and a 55-year old Caucasian male. Both patients were referred to the staff chiropractor at the facility by medical physicians for the management of back pain and related symptoms.

Inclusion criteria for patients to be incorporated in this analysis were clinical signs and symptoms of pain that were initially thought to be biomechanical in origin, but where the patient was eventually found to have a malignancy in the general region of the initial complaints. The malignancy did not have to be responsible for the patient's initial complaints, however.

The patients reported upon in this manuscript are now deceased and their consent to publish this report could therefore not be obtained.

High-velocity low-amplitude (HVLA) manipulation along with along with various physical medicine procedures and therapeutic exercise was the management regimen used in these patients. The course of chiropractic care was nine weeks for the 54-year old female patient and four weeks for the 55-year old male. Each patient was seen 1–2 times per week during the course of care.

The patients in this retrospective case series were treated with HVLA manipulation and other physical medicine interventions. The patients in this cohort were not subjected to any additional, non-routine clinical procedures as part of the chiropractic management protocol. The male patient did have trigger point injections into several intercostal muscles after failing to respond to the care provided by the chiropractor.

Outcomes were based on improvement in clinical signs and symptoms, as well as the need for future intervention. Outcomes were classified into 3 categories: significant improvement, moderate improvement, and no change.

Significant improvement was defined by at least a 90% resolution of the pain syndrome, based on pre and post treatment visual analog scale (VAS) (0–100) measures and with the ability to perform all normal activities of daily living (ADL) after care, as reported by the patient. Such patients would require no further conservative or surgical intervention. Moderate improvement was defined as between a 50–90% reduction of the pain syndrome, as measured by the difference between pre and post treatment VAS scores. In addition, such improvement would include only mild restriction in ADLs after care, as reported by the patient. The need for further conservative care was warranted, but no surgical intervention was required. A patient who did not exhibit at least a 50% improvement was placed in the no change category. The presence of any adverse side-effects resulting from the therapy prescribed in these cases was based on review of the patients' clinical records.

Each patient was treated with HVLA manipulation, spray and stretch, massage and therapeutic exercise. The female patient was treated in the lumbar spine with lateral decubitus manipulation and the male patient was treated with prone manipulation of the thoracic spine.

Each patient initially responded positively to therapy, including a decrease in VAS scores and increased subjective functionality as measured by increases in the ability to perform normal ADLs.

The first patient was referred by her primary care physician for evaluation and management of non-organic biomechanical low back pain after a course of NSAIDs and pain medications. She had had moderate pain that was persistent for several months before admission. She demonstrated significant improvement with two weeks of chiropractic care as measured by progressively decreasing VAS scores. After the initial course of care which consisted of HVLA and continuous flexion distraction, she began having increasing low back and pelvic pain. The pain was different than at initial presentation and she appeared ill/gaunt. At this time, she also reported to the chiropractor managing her case that she had noted a 23 pound weight loss within the previous month.

**Figure 1:**
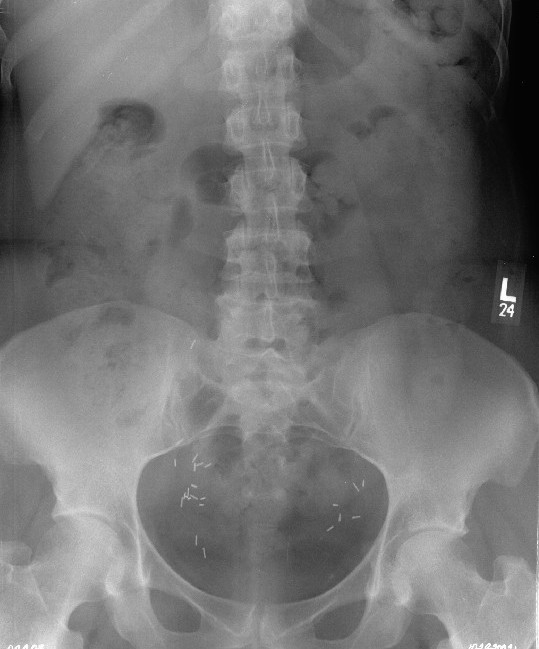
**AP lumbar spine radiograph for patient #1**. This film was interpreted as normal.

**Figure 2:**
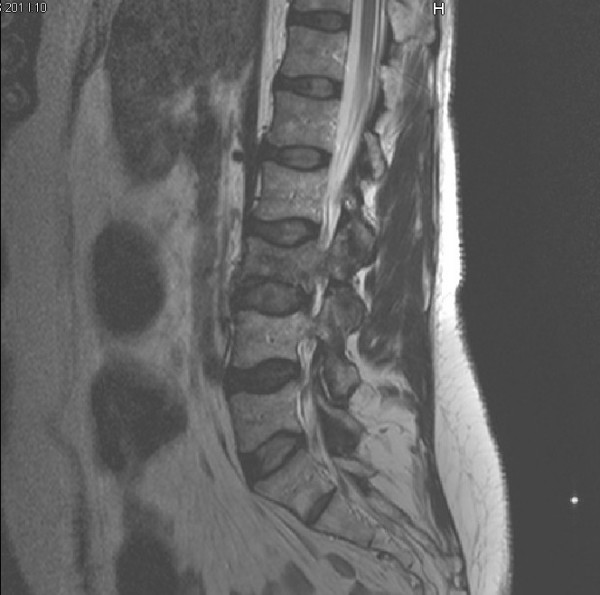
**Sagittal T1-weighted lumbar Spine MRI for patient #1**. There is low signal intensity destruction within the vertebral body extending into the right neural arch at the L3 level. In addition, collapse of the L3 vertebral body is identified.

**Figure 3:**
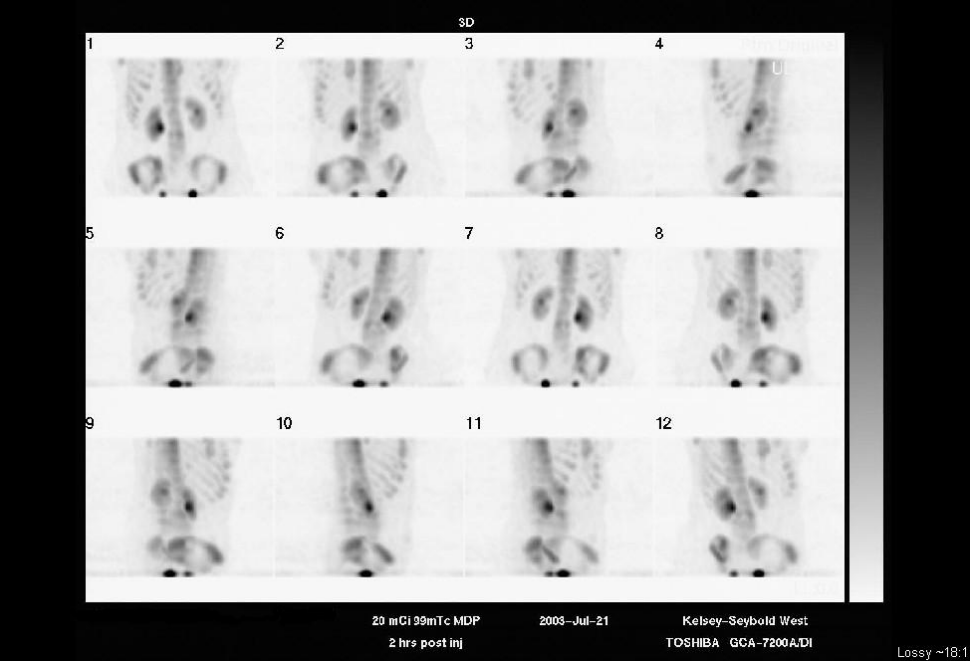
**Radionuclide bone scan of lumbar spine for patient #1**. This scan of the lumbar spine demonstrates only minimally increased uptake at the L3 vertebral level, primarily in the vertebral body, but also possibly in the region of the pedicles.

**Figure 4:**
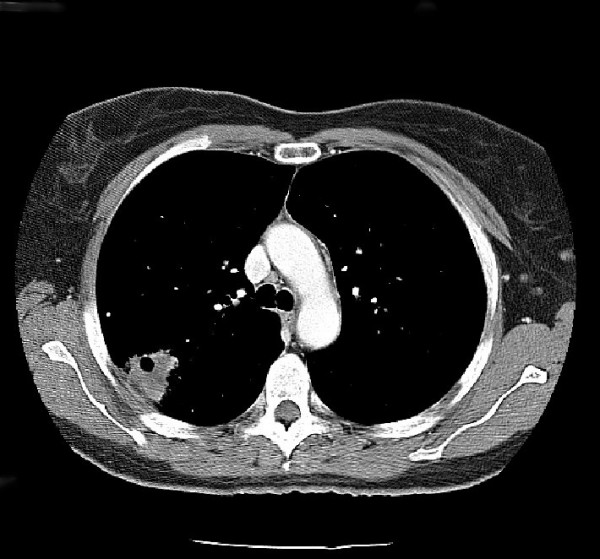
**Contrast-enhanced CT scan of the chest for patient #1**. A large cavitating lesion was identified in the right upper lobe with pleural involvement and likely extension into the chest wall. In addition, right hilar lymphadenopathy was suspected. This was the primary malignant lesion in this patient.

Plain film radiographs were obtained and read as normal. Figure 1 is the anteroposterior lumbar radiograph. MRI of the lumbar spine was then obtained. It was suspicious for an aggressive-looking lesion, suggestive of malignancy, at the L3 level. (Figure 2) Interestingly, a radionuclide bone scan demonstrated only mild uptake at the L3 level. (Figure 3) Blood work, including a complete blood count and serum chemistries, was normal. Computerized tomography (CT) scans of the chest (Figure 4) were obtained. (Figure 4) and demonstrated a large cavitating lesion in the posterior aspect of the right upper lobe with probable pleural involvement likely representing the primary lesion. In addition, there was also suspicion of lymphadenopathy in the right hilum. An abdomen CT scan was also obtained, and was interpreted as normal. Primary small-cell bronchogenic carcinoma with skeletal metastases were identified.

In the second case, the male presented with mid-back pain in the peri-scapular area bilaterally and without any radiating symptoms. An initial 6-week course of treatment resulted in no change by measure of VAS scores and his ability to perform normal ADLs. He had previously been treated with HVLA for similar pain, which had resolved his complaints.

However, he returned after two months with the similar mid-back pain and now, pain along the mid-right anterior-axillary line. The anterior-axillary pain was new, more severe as measured subjectively and with VAS, respectively. Based upon follow up examination he was referred for trigger point injections and was examined and treated by a physical medicine and rehabilitation (PMR) physician. His examination included chest film radiographs and treatment was focused in intercostals trigger point injections.

**Figure 5:**
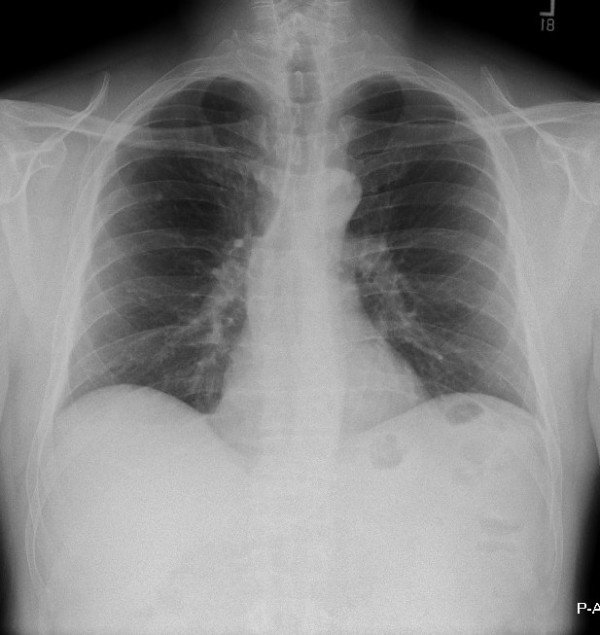
**PA chest radiograph for patient #2**. This film was interpreted as normal.

His chest film was read as normal. (Figure 5)

**Figure 6:**
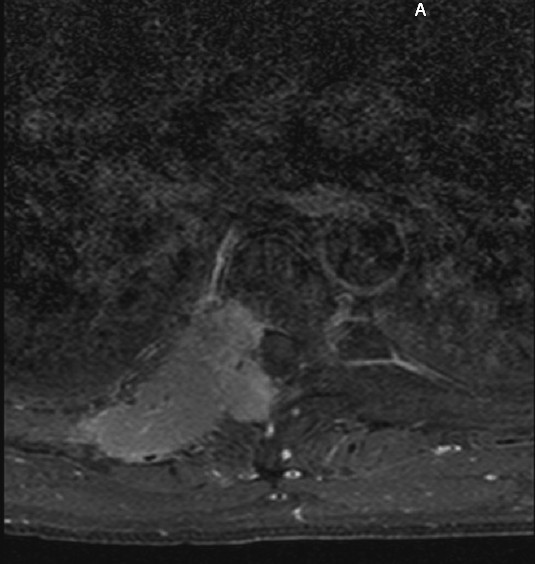
**Contrast-enhanced T1-weighted axial MRI of the T6 level for patient #2**. This slice demonstrates a large multi-lobulated mass involving the vertebral body and neural arch with extension into the spinal canal, intervertebral foramen and chest wall. It represents one focus of metastases from a primary renal cell carcinoma.

He returned after re-evaluation and treatment by the PMR physician. He now had posterior chest wall/rib pain at the 5–8 ribs on the right. A thoracic spine MRI was ordered to evaluate for the possible presence of soft tissue and/or disk injuries, whereupon high signal intensity lesions of the spine and ribs were discovered. Figure 6 is a post-contrast T1-weighted axial image demonstrating a large mass invading the T6 vertebra, spinal canal and regional chest wall structures. Referral to his primary care physician, and eventually the oncology service, was made for management of his malignancy, whereupon all of these lesions were found to be metastases as the result of a primary renal cell carcinoma.

Current guidelines and best practices initiatives suggest that radiography in patients with apparently uncomplicated/non-organic biomechanical back pain without "red flags" is not indicated. The initial work-up of patients in these circumstances includes a complete history and physical examination. The Agency for Health Care Policy and Research(AHCPR), now called the Agency for Healthcare Research and Quality, published guidelines for the workup of patients with back pain. [17] They suggest that the use of plain film radiography should be restricted to those patients who have red flags from clinical history or physical examination. Table 1 outlines the typical red flags.

**Table 1:** Red Flags [17]

**HISTORY**	**PHYSICAL EXAMINATION**
Cancer	Saddle anesthesia
Unexplained weight loss	Loss of anal sphincter tone
Immunosuppression	Major motor weakness in lower extremities
Prolonged use of steroids	Fever
Intravenous drug use	Vertebral tenderness
Urinary tract infection	Limited spinal range of motion
Pain that is increased or unrelieved by rest	Neurologic findings persisting beyond one month
Fever	
Significant trauma related to age (e.g., fall from a height or motor vehicle accident in a young patient, minor fall or heavy lifting in a potentially osteoporotic or older patient or a person with possible osteoporosis)	
Bladder or bowel incontinence	
Urinary retention (with overflow incontinence)	

Deyo and Diehl suggested a number of indications for radiography in patients with low back pain, which differ from the AHCPR Guidelines. [18] Their suggestions were formulated prior to the AHCPR report. An adaptation of their criteria is outlined in Table 2. Wyatt and Schultz suggested similar criteria in 1987. [19]

**Table 2:** Selective Indications for Radiography in Acute Low Back Pain [18]

Age > 50
Significant trauma (fall from more than 10 feet)
Progressive neuro-motor deficits
Unexplained weight loss (10 lb in six months)
Suspicion of ankylosing spondylitis
Drug or alcohol abuse
History of malignancy
Use of corticosteroids
Fever
Recent visit (within 1 month) for same problem with no improvement
Patient seeking compensation for back pain(work-related injury)

The number, sequence and type of standard views for an examination should be problem-oriented and have clinical efficacy in terms of impact on treatment or prognosis. [19,20] A patient should never be exposed to unnecessary radiation. Areas of exposure as well as the number of exposures should be kept to a minimum. Routine and/or repetitive radiographic examinations for demonstration of subluxations or as a screening procedure (e.g., pre-employment) are not considered appropriate diagnostic strategies. [19,21,22]

In the first patient presenting with spine pain in this case series, her initial response was quite favorable. It was not until she began to demonstrate red flag signs (eg, unexplained weight loss and a recent visit for the same problem with no improvement) that radiographs were performed. Those radiographs were normal. It was decided that with the presence of such significant red flag signs that she should undergo advanced imaging, in this case an MRI, that revealed an underlying malignancy, which was likely not the primary cause of her initial back pain, which responded to conservative care as outlined above. A best practices approach was utilized in this patient and had no substantial negative effect on her outcome.

In the second case, the gentleman presented with, what appeared to be to the medical and chiropractic doctors, non-organic biomechanical pain in the thoracic spine. Radiography was not initially performed. It was not until the patient failed to respond to conservative care that radiography was performed and, in this case was interpreted as normal. The patient again failed to respond to a different course of conservative care, whereupon MRI was performed. Once again, a best practices approach was utilized and did not adversely affect the outcome for this patient.

Very often, apparently otherwise healthy patients present to doctors of chiropractic with neuromusculoskeletal symptoms that appear to be non-organic biomechanical or uncomplicated in etiology. Often, as a part of the initial workup of these patients, plain film radiography is performed and many times, as was the case in the two patients in this case series, the plain films are interpreted as being normal.

In a study designed to assess the use of radiography, Harger, et.al. discovered that 74% of the chiropractors have radiographic facilities in their offices. The most common reasons listed for performing radiography included contraindication to manipulation screening (71%), pathological diagnosis (63%), biomechanics and posture (51%) and medicolegal protection (27%). [3] These findings are contradictory to what current best practices and clinical guidelines suggest. In Canada, Ammedolia, et.al. had similar findings, where 63% of chiropractors suggested that they would use radiography on patients with uncomplicated acute low back pain lasting 1 week. In addition, 68% stated that radiographs were useful in the diagnostic evaluation of patients with acute low back pain lasting less than 1 month. They conclude that "most reasons given for use of radiography in this patient population are not supported by existing evidence." [5]

Physicians often suggest that screening for serious pathology is an acceptable reason for performing radiographs on most all, if not all patients, who present with spine pain. Finding an occult malignancy is an oft cited reason for this practice. Deyo and Diehl evaluated 1,975 walk-in patients with back pain. Of those patients, 13 (0.66%) eventually were diagnosed with malignancy as the cause of their back pain. Age of 50 years or greater, previous history of cancer, failure to improve within one month and anemia were some of the primary findings that were associated with malignancy in their patient cohort. They developed a diagnostic algorithm that combined history and physical examination findings with erythrocyte sedimentation rate that would have reduced the percentage of patients who were radiographed to 22%, while still uncovering all of the malignancies in their patients. [23]

In some patients, as was the case in this investigation, plain film radiographs are normal when there may be an underlying aggressive pathology. Deyo and Diehl suggest that in patients who have negative radiographs, but in whom the findings noted above are seen, further workup, including advanced imaging, would be worthwhile. [23] In our two patients, Deyo and Diehl's suggestions proved very useful and found what were likely occult malignancies.

Even in light of rising health care costs, some providers suggest that patients who have back pain expect radiography as a part of the clinical services provided by physicians. Deyo, et. al. examined the psychological, functional, and financial consequences of omitting spine films for patients with back pain where the patients had only minimal risk of having underlying aggressive disease. Their patients were divided into two groups. One group received immediate radiography of the area of chief complaint upon admission and the other group received a brief educational intervention about back pain and radiography. Radiography would only be performed on patients in the second group for failure to improve. Initially, 73% of the group who had immediate radiography believed that people with back pain should have an x-ray, while only 44% of the education group had the same thoughts. After three months, only 31% of those patients in the education group had received radiography. Radiology charges in the second group were still far less than those of the group with immediate radiography. Of particular importance is the fact that no aggressive spinal disease was missed, and outcomes for the two groups were the same. They conclude that, "eliminating or delaying spine films need not cause anxiety, dissatisfaction, or dysfunction. This strategy may modify future expectations of roentgenography use and reduce health care costs." [24] Kendrick, et.al. arrived at similar conclusions regarding patient satisfaction and radiography. [25] Kendrick, et.al., in another study of radiography in the primary investigation of back pain concluded that radiography of the lumbar spine in low back pain patients was not associated with improved patient functioning, severity of pain, or overall health status. [26] Kerry, et.al. had almost identical findings and conclusions. In addition, the diagnostic yield of plain film radiography, as evidenced by these studies is relatively low.

## Conclusion

In these two cases, standard evidence-based medicine guidelines and best practices were utilized in making clinical decisions about the care for these patients, without any unwanted or adverse side effects. Although both patients were eventually diagnosed with malignancy, this approach did not significantly alter the appropriate long-term management of these patients who presented to a chiropractor with neuromusculoskeletal signs and symptoms. Interestingly, manipulation provided some positive outcomes in these patients, suggesting that these patients had both uncomplicated/non-organic biomechanical spine pain along with malignancies. In both cases, plain film radiographs were initially thought to be of little help, as there was a low index of suspicion for cancer. Plain film radiographs were eventually obtained and were essentially normal without indication of malignancy in either case.

The judicious use of MRI was an effective procedure when investigating recalcitrant neuromusculoskeletal pain in the patients in our series.

## Competing interests

The author(s) declare that they have no competing interests.

## Authors' contributions

RKP conceived of the study, and participated in its design and coordination and helped to draft the manuscript. LHW participated in the study design and records review, helped to draft the manuscript, and was the corresponding author. Both authors read and approved the final manuscript.
